# The behavioral response of prey fish to predators: the role of predator size

**DOI:** 10.7717/peerj.3222

**Published:** 2017-04-20

**Authors:** Zhong-Hua Tang, Qing Huang, Hui Wu, Lu Kuang, Shi-Jian Fu

**Affiliations:** Laboratory of Evolutionary Physiology and Behavior, Chongqing Key Laboratory of Animal Biology, Chongqing Normal University, Chongqing, China

**Keywords:** Anti-predation strategy, Body size, Swimming activities, Gape size, Vigilance, Channidae, Cyprinidae

## Abstract

Predation is one of the key factors governing patterns in natural systems, and adjustments of prey behaviors in response to a predator stimulus can have important ecological implications for wild fish. To investigate the effects of predators on the behavior of prey fish and to test whether the possible effects varied with predator size, black carp (Mylopharyngodon piceus) and snakehead (Channa argus) (a size-matched predator treatment with a similar body size to prey fish and a larger predator treatment with approximately 2.7 times of the body mass of prey fish) were selected to function as prey and predator, respectively. Their spontaneous activities were videorecorded in a central circular arena surrounded by a ring holding the stimulus fish. The distance between prey and predator fish was approximately 200% of the distance between two prey fish, which suggested that black carp can distinguish their conspecifics from heterospecifics and probably recognize the snakehead as a potential predator. The prey fish spent substantially less time moving and exhibited an overall shorter total distance of movement after the size-matched or large predator was introduced, which possibly occurred due to increased vigilance or efforts to reduce the possibility of detection by potential predators. However, there was no significant difference in either distance or spontaneous activities between two predator treatments. These findings suggested that (1) an anti-predator strategy in black carp might involve maintaining a safe distance, decreasing activity and possibly increased vigilance and that (2) the behaviors of prey response to predators were not influenced by their relative size difference.

## Introduction

Predation is one of the central factors governing patterns in natural systems (Sih, Englund & Wooster, 1998), and the prey behavior is expected to change as a result of predation (Diehl & Eklöv, 1995; Ryan et al., 2012; Fu et al., 2015a; Fu et al., 2015b). For example, it has long been found that the chemical and visual cues of predators elicited alterations in space use and decreased foraging and exploration swimming activity in juveniles of fish species such as sticklebacks (*Gasterosteus aculeatus*), perch (*Perca fluviatilis*) and rainbow trout (*Oncorhynchus mykiss*) (Milinski & Heller, 1978; Fraser & Gilliam, 1992; Diehl & Eklöv, 1995; Brown & Smith, 1998; Lehtiniemi, 2005; Kopack et al., 2015). However, in fish species such as bluegill sunfish (*Lepomis macrochirus*), a predator stimulus showed the opposite effects, i.e., no change in spatial position or refuge seeking (Urban, 2007; Oplinger, Wahl & Nannini, 2011; Albecker & Vance-Chalcraft, 2015). The difference might be due to differences in morphological, ecological characteristics, nutritional status and anti-predator strategy of the prey as well as the hunting strategies of different predators (Peacor, 2002; Oplinger, Wahl & Nannini, 2011; Lönnstedt et al., 2012). Furthermore, it has been found that whether a prey can escape from its predator largely depends on relative body size difference between them which is mainly imposed by morphological restriction of the predator’s mouth width (also known as gape size limitation) (Dörner & Wagner, 2003). Thus, body size effect has attracted the attention in research and is usually regarded as one of the critical characteristics correlated with prey survival during predatory encounters (Holmes & McCormick, 2010; Rodgers, Downing & Morrell, 2015). Therefore, the behavior of prey to its predator will likely be quite different if the relative body size difference between them changes. Thus, examining behavioral responses of prey fish to its predator and the possible impact of relative body size difference between them on these responses is very important. To date, research on the relative sizes of prey and predator has attracted increasing attention, but studies have mainly considered the survival indicators of prey fish. The present study is the first to explore the behavioral response of a prey fish to its predator as well as the effect of the body size of predators on the behavioral response of the prey.

To achieve these goals, we selected the black carp (*Mylopharyngodon piceus*), a benthic cyprinid fish species mainly feed on aquatic mollusks, shrimp and aquatic insects, as the prey species. The snakehead (*Channa argus*), a typical benthic piscivorous predator widely used in fish behavior research (Wolf & Kramer, 1987; Liu et al., 2016), was used as the predator species. The black carp is widely distributed in rivers and lakes throughout eastern Asia and prefers to live in still or slowly flowing water with abundant aquatic zoobenthos, whereas snakehead is distributed in rivers, lakes and ponds throughout Asia and Africa and prefers to live in still or slowly flowing water with abundant aquatic vegetation. Thus, both fish species are distributed in overlapping natural habitats, and juvenile black carp are preyed upon by the snakehead (Böhme, 2004). To fulfill our goal, we paired black carp with snakeheads that either had a similar body size or approximately 2.7 times of the body mass of black carp (this difference ensured the snakeheads could successfully prey on the black carp according to our pilot experiment) and a control treatment with black carp paired with conspecifics. Then, we videorecorded the activities of prey with or without the presence of predators of different sizes and evaluated their response by the distance between fish, the percentage of time spent moving, swimming speed, and the total distance moved.

## Materials and Methods

### Experimental fish and acclimation

Both juvenile black carp (predator naïve) and snakeheads were collected from local fishery on May, 2015. Prior to the experiment, the fish were housed in laboratory tanks with recirculating water for 2 weeks at a water temperature of 25 ± 0.5 °C. During this period, the black carp were fed to satiation with a commercial floating diet (Tongwei Company, Chengdu, China; composition: 41.2 ± 0.9% protein; 8.5 ± 0.5% lipid; 25.7 ± 1.2% carbohydrate and 12.3 ± 0.4% ash), and the snakeheads were fed fresh black carp once daily at 08:00. The food-residue and feces were cleared using a siphon 1 h after feeding. The water was constantly aerated to ensure that dissolved oxygen was always at least 90% saturation. The photoperiod cycle was 12 h light: 12 h dark. The experiment measurements were performed from 8:00–18:00 after two weeks of pre-acclimation. This study was approved by Animal Care and Use Committee of the Key Laboratory of Animal Biology of Chongqing (Permit Number: Zhao-20150225-01) and performed in strict accordance with the recommendations in the Guide for the Care and Use of Animal at the Key Laboratory of Animal Biology of Chongqing, China.

### Experimental setup

**Figure 1:**
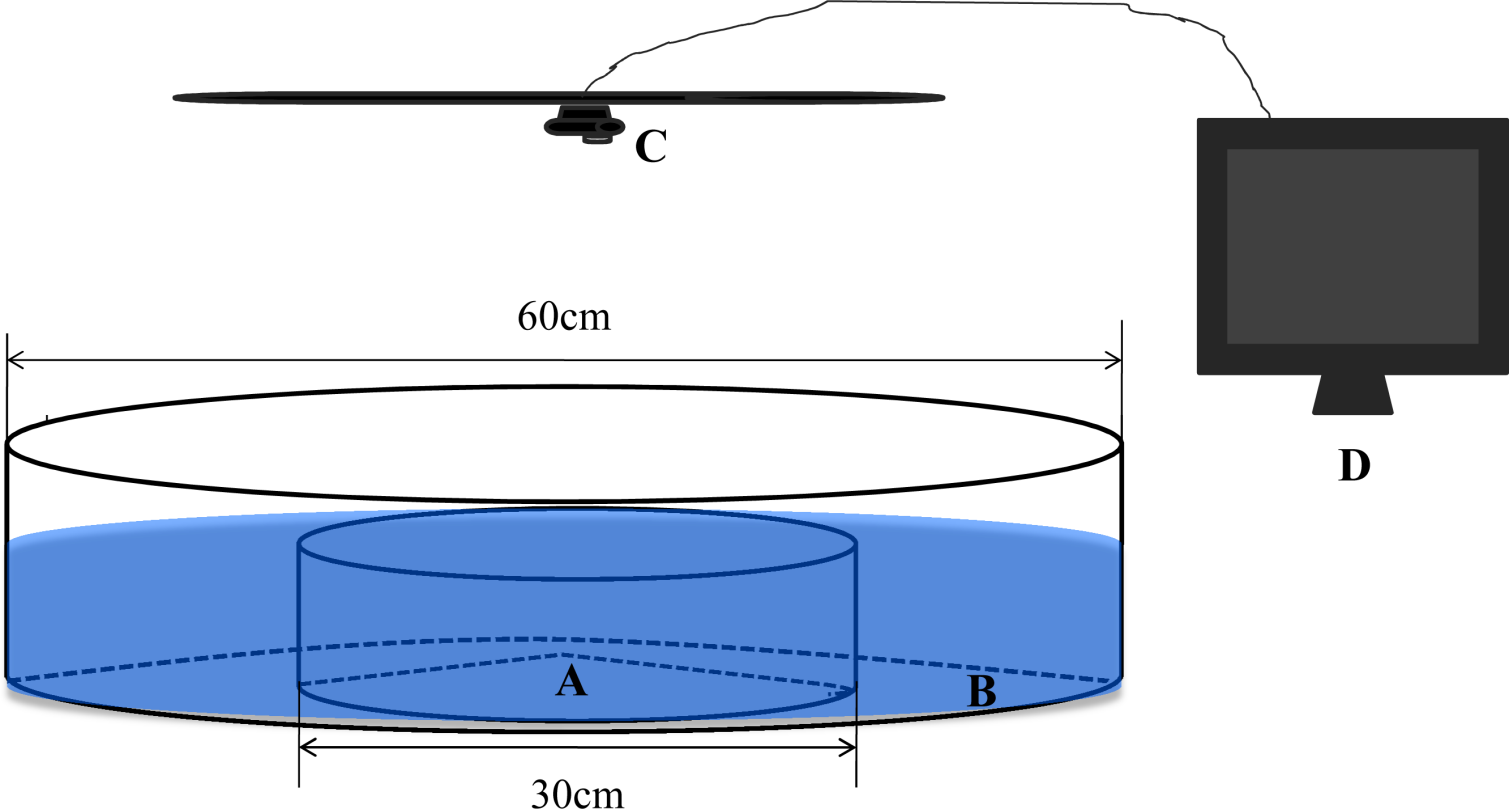
The experimental observation setup used in the present study. (A) Central arena. (B) Ring area. (C) Camera webcam. (D) Monitor.

We constructed a circular observation chamber with a diameter of 60 cm from 1 cm-thick, opaque, white acrylic (Fig. 1). This chamber consisted of a central circular arena (A, diameter = 30 cm) and a concentric annular ring (B, diameter = 60 cm) separated by 0.2 cm of transparent acrylic. To facilitate transmission of physical and chemical cues between the separate areas, holes with a diameter of approximately 0.3 cm were drilled in the transparent barrier ring and evenly distributed at a density of approximately 200 m^−2^. The depth of the water for all trials was maintained at 10 cm (Hoare et al., 2004). Shadowless illumination of the experimental setup was provided by incandescent lamps. A camera webcam (C, Logitech Pro 9000) connected to a remote monitor (D) was installed directly above the observation chamber. To minimize disturbance by observers during the experiments, the experimental tank was surrounded with opaque canvas.

### Experimental protocol, methods and parameter calculation

**Figure 2:**
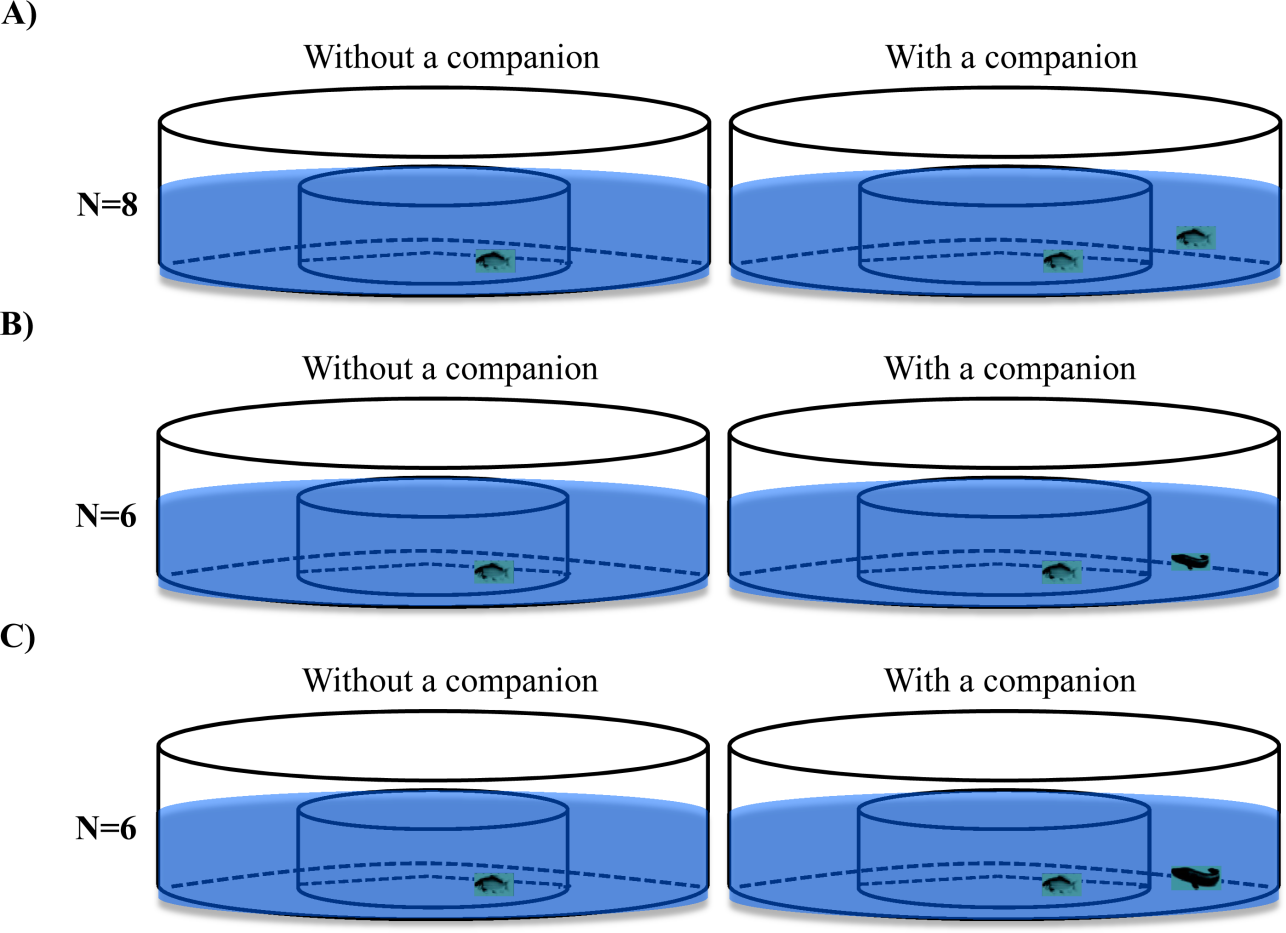
The schematic diagram of the experimental design used in the present study. The fish in the control treatment (A) were black carp in both the central circular arena and ring area, whereas in the size-matched (B) and larger predator (C) treatments, the fish in the central arena were black carp, while the fish in the ring area were size-matched or larger snakehead.

Two experimental treatments (i.e., size-matched predators and larger predators) and a control treatment (size-matched conspecifics) were videorecorded during the experiment (Fig. 2). Black carp with a similar body size (body mass: 2.61 ± 0.10 g, standard body length: 5.55 ± 0.06 cm, *N* = 28) were randomly assigned among three experimental groups, and snakehead with two different body sizes (body mass: 2.57 ± 0.12 g, standard body length: 5.86 ± 0.12cm, *N* = 6; body mass: 7.01 ± 0.22 g, standard body length: 7.88 ± 0.15 cm, *N* = 6) were assigned to either the size-matched predator treatment or larger predator treatment. In the control treatment, a black carp was individually transferred to the central arena of the experimental setup and videorecorded (15 frames per second) for 1 h. Then, another black carp was transferred to the ring area and videorecorded for another hour (*N* = 8). In the experimental treatments (i.e., the size-matched predator treatment and large predator treatment), the procedure was the same as for the control treatment, except that a snakehead was placed in the ring area instead of a black carp (*N* = 6).

The videos were converted from .wmv to .avi format with Format Factory (http://format-factory.softonic.cn). Then, the centroid-based trajectories of the fish and the coordinates of each frame with the range of 10 min before and 10 min after introduction of another fish were obtained using idTracker (Pérez-Escudero et al., 2014). The relative size between the pixels and entities was used to calculate the real coordinates of the experimental fish. The parameters for describing spontaneous activities and their interactions were calculated with the formulas listed below.

Distance (cm) between centroids of two fish:

(1) \begin{eqnarray*}D(t)=\sqrt{({x}_{1}(t)-{x}_{2}(t))^{2}+({y}_{1}(t)-{y}_{2}(t))^{2}}\end{eqnarray*}

where *x*(*t*) and *y*(*t*) are the coordinates of the centroid position of two fish in the horizontal plane at time *t*.

Spontaneous swimming speed while moving (cm s^−1^):

(2) \begin{eqnarray*}v(t)=\sqrt{(x(t)-x(t-1))^{2}+(y(t)-y(t-1))^{2}}/dt\end{eqnarray*}

where *x*(*t*) and *y*(*t*) are the coordinates of the centroid position of either prey or predator in the horizontal plane at time *t*, *x*(*t* − 1) and *y*(*t* − 1) are the coordinates in the previous frame, and *dt* is the time interval between successive frames (one-fifteenth, i.e., 0.067 s). Fish were defined as moving when the values were higher than 1.75 cm s^−1^.

Percent time moving (%) of individual fish:

(3) \begin{eqnarray*}Percent time moving= \frac{{T}_{2}}{{T}_{1}} \times 100\text{%}\end{eqnarray*}

where *T*_1_ is the duration of observation and *T*_2_ is the time spent moving (i.e., swimming speed higher than 1.75 cm s^−1^).

Total distance moved (cm) was the product of swimming speed and percentage time of movement.

### Data analysis

SPSS Statistics 17 (IBM, Armonk, NY, USA) was used for data analysis. All values are presented as the means ± SE, and *P* < 0.05 was considered statistically significant. The difference in the distance between the two fish among the control and two treatments was assessed using one-way analysis of variance (ANOVA). The effects of experimental treatments (control, size-matched and large predator) and predator exposure (before and after introduction of a companion) were tested by linear mixed model analysis with fish ID as random factor. A Duncan test or paired *t*-test was performed if a statistical assessment of the difference among three treatment groups or between those with or without a companion was necessary. All data were tested for normality by One-Sample Kolmogorov–Smirnov Test.

## Results

### The distance between fish

**Figure 3:**
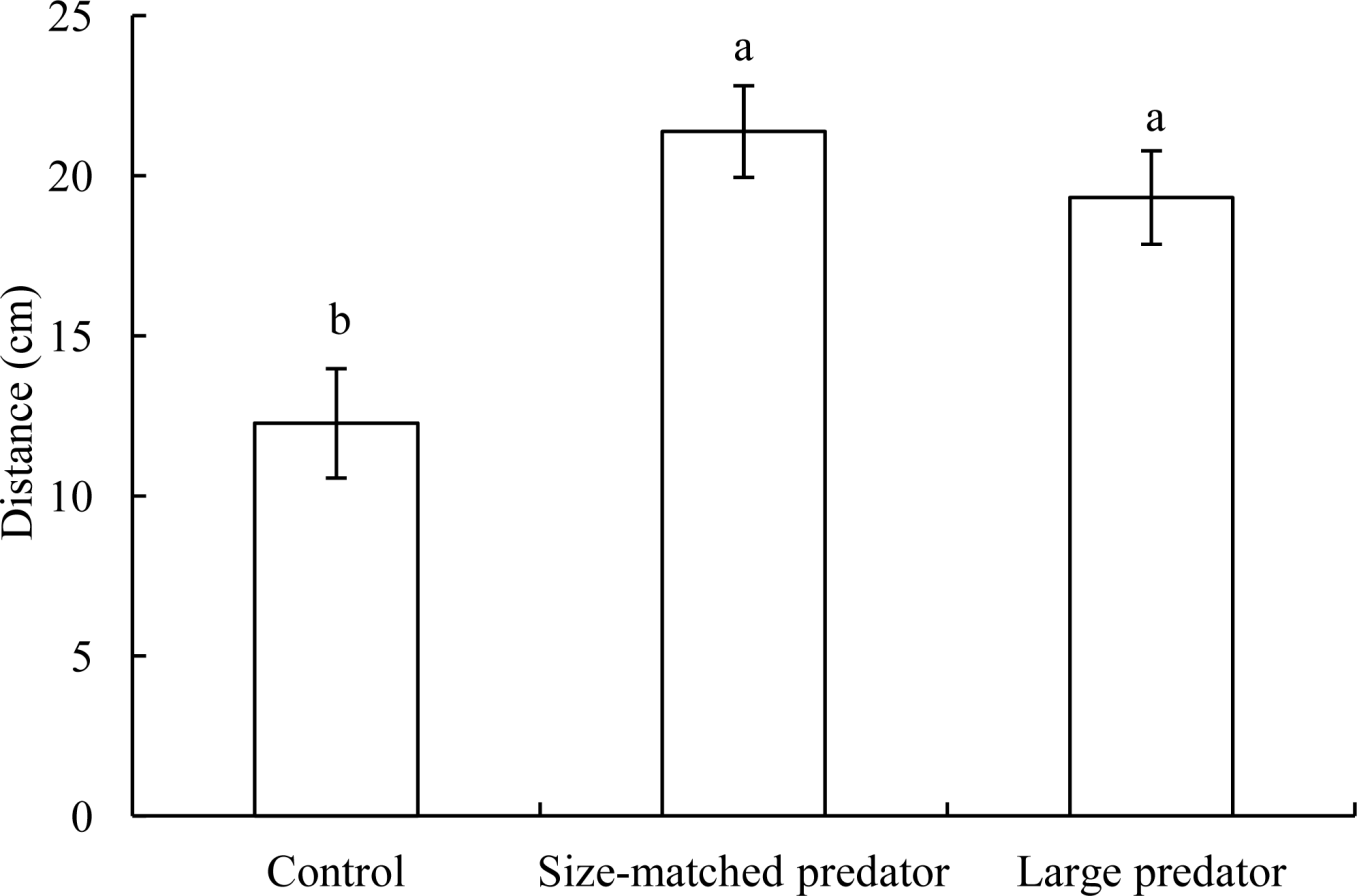
The distance between two individuals (mean ± S.E., see Fig. 2 for replicates) in the control treatment and the experimental treatments. (A) and (B) indicate significant differences in distance between the control and predator treatments (*P* < 0.05).

In the control treatment, the distance between black carp individuals in the central arena and ring area was approximately 10 cm (Fig. 3). However, when a snakehead was placed in the ring area (whether it was size-matched or larger), the distance between prey and predator fish significantly increased to approximately 20 cm (*F*_2,17_ = 9.599, *P* = 0.002), whereas there was no significant difference in the distance between the size-matched and larger predator treatments.

### Activity of the black carp

**Figure 4:**
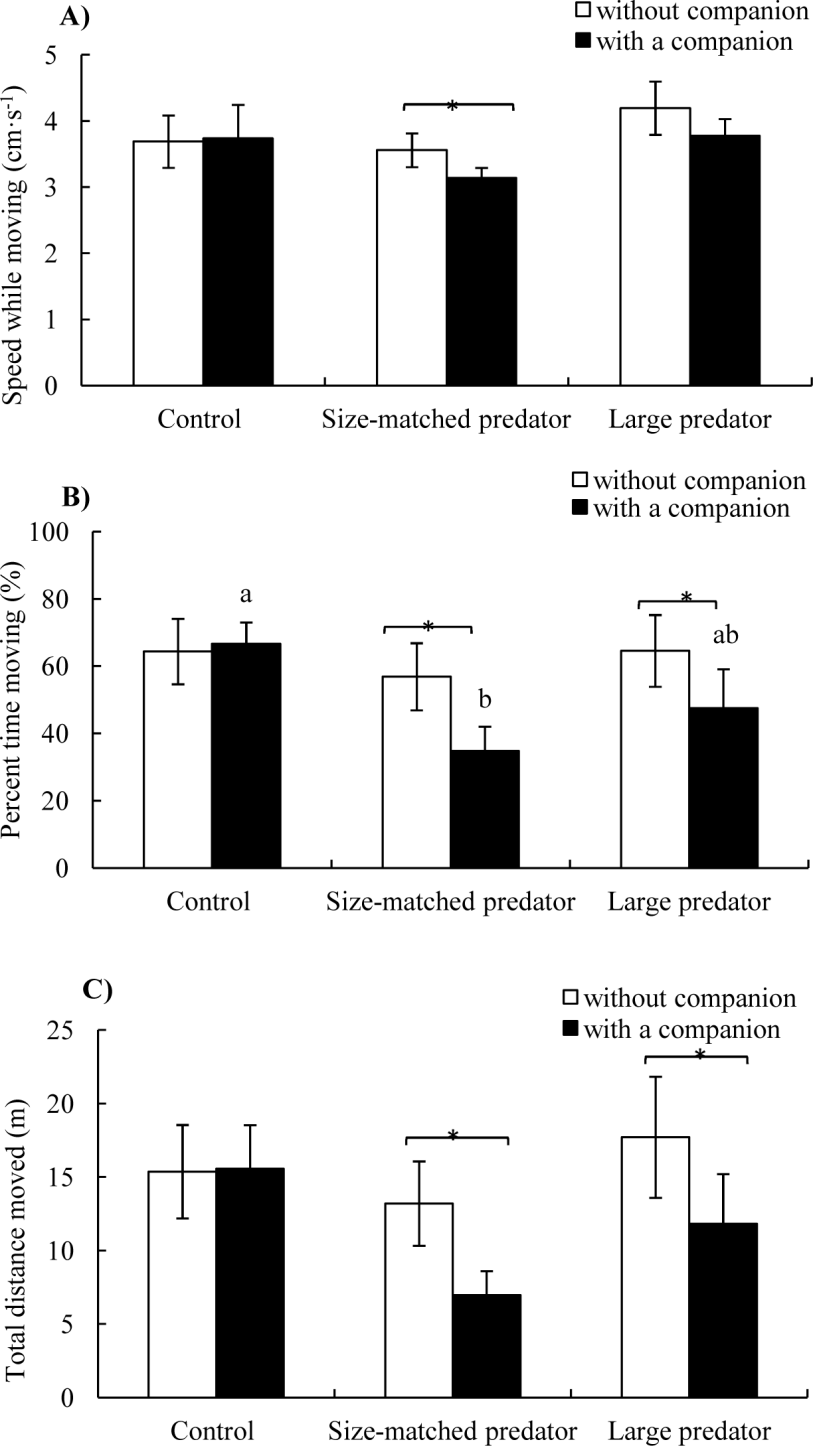
The speed while moving (A), percent time moving (B) and total distance moved (C) of the black carp in the control and experimental treatments during without and with a companion (mean ± S.E., *N* = 6). * indicates significant differences between without companion and with a companion within the prey; a and b indicate significant differences between the control and predator treatment during with a companion conditions (*P* < 0.05).

*Speed while moving:* Neither treatment (*F*_2,17_ = 0.981, *P* = 0.395) nor companion (*F*_1,17_ = 1.020, *P* = 0.327) showed a significant effect on speed while moving (Table 1 and Fig. 4A).

**Table 1:** The effect of predator treatment on the activities of prey based on a linear mixed model using fish ID as the random factors.

	Treatment	Companion	Treatment × Companion
Speed while moving (cm s^−1^)	*F*_2,17_ = 0.981	*F*_1,17_ = 1.020	*F*_2,17_ = 0.392
	*P* = 0.395	*P* = 0.327	*P* = 0.682
Percent time moving (%)	*F*_2,17_ = 1.414	*F*_1,17_ = 7.008	*F*_2,17_ = 2.820
	*P* = 0.270	*P* = 0.017	*P* = 0.088
Total distance moved (cm)	*F*_2,17_ = 0.986	*F*_1,17_ = 7.955	*F*_2,17_ = 2.406
	*P* = 0.393	*P* = 0.012	*P* = 0.120

*Percent time moving:* There was no significant difference of percent time moving among three groups without companion (i.e., before introduction of the second fish), whereas the percent time moving of the fish in the control treatment was significantly higher than size-matched predator treatment after introduction of a companion fish (*P* < 0.05). There was no significant difference before and after introduction of the companion fish in the control group, whereas the percent time moving decreased significantly after the introduction of the companion fish in both the size-matched predator and large predator groups (*P* < 0.05) (Fig. 4B).

*Total distance moved:* Total distance moved showed the same trend as percent time moving except that there was no significant difference among the three groups after the introduction of the companion fish (Fig. 4C).

## Discussion

### The effect of a predator on the distance between fish

Black carp can distinguish conspecifics from snakeheads, as suggested by the distance between them. The distance between black carp in the control treatment was approximately 10 cm, while the distance between black carp and snakehead in both predator treatments was approximately 20 cm. This suggests that black carp can distinguish their conspecifics from heterospecifics. This pattern might be due to an attraction between black carp rather than avoidance of the snakehead, which is reasonable because black carp prefers to live in groups (Fu et al., 2016). Furthermore, the distance between black carp and snakehead (i.e., 20 cm) is similar to distance expected in a random distribution (i.e., half the diameter of the center arena (15 cm) plus half the width of the ring (7.5 cm) is 22.5 cm). However, it is also possible that black carp recognize snakehead as predators and therefore maintain a safe distance. The first reason for such conclusion is that both black carp and snakehead preferred to stay close to the edges of arena rather than open area thus the distance between two fish individuals should be much shorter than expectation based on randomly distributed positions. Secondly, a previous study on schooling behavior of omnivorous cyprinid fish species found that heterospecific individual pairs showed a similar distance to that of conspecific pairs (Z-H Tang, 2016, unpublished data). Lastly, behavioral adjustments of black carp to the presence of snakeheads, i.e., the reduction of activity after introduction of snakehead further confirmed such conclusion. Interestingly, a recent study on the interactions between snakehead as predator and rose bitterling (*Rhodeus ocellatus*) as prey also found that the prey fish preferred to stay a similar distance away from the predator, i.e., 20 cm (Hu, Nie & Fu, 2015). As cyprinids, black carp and rose bitterling may have similar anti-predator strategies. Furthermore, studies on fish species other than cyprinids (e.g., pike, *Esox lucius*; three-spined stickleback, and Nile tilapia, *Oreochromis niloticus*) also found that prey fish were capable of detecting predators through chemical and visual cues, and thus they maintained a greater distance from predators, spent more time in shelter areas and decreased their swimming activity when exposed to natural predators (Lehtiniemi, 2005; Freitas & Volpato, 2008). Maintaining a safe distance from predators thus ensured enough time for response to and escaping from a potential attack, which is a vital strategy for prey fish in nature. Since the black carp used in the present study is predator-naive fish, it was also possible that the black carp regarded the snakehead as non-conspecific individuals and a potential risk, and the behavior that maintained a safe distance from hetero-species may likely decrease some unknown cost, such as the competition for space, food and other resources (Ahmad et al., 2010; Ng & Tan, 2010; Winandy & Denoël, 2013).

It has long been assumed that the relative size between prey and predator is a vital factor influencing the results of the activity of prey interaction, i.e., relatively smaller prey may have a lower escape capacity and are easier to catch (Congdon et al., 1999). Thus, we might expect that black carp exposed to larger predators would have a longer safety distance from predators. A similar distance between two treatments might suggest that either snakehead showed no effect on the position of black carp as mentioned above or that the distance itself is more important than other factors that might ensure enough response time for escape. Studies of cyprinid fish species such as qingbo (*Spinibarbus sinensis*) and pale chub (*Zacco platypus*) found that the latency of escape response was more important than the swimming capacity in anti-predator strategies, as indicated by maximum speed or acceleration speed (Fu et al., 2015a; Fu et al., 2015b; Liu et al., 2016). Another explanation for this result might be the limitation of the perceptual capacity in black carp, i.e., it may be difficult to obtain information about predators beyond a distance of 20 cm. It is worth noting that rose bitterling, which are approximately one-sixth the body size of snakehead, also maintained a similar safety distance from its predator, which also supports our conclusion (Hu, Nie & Fu, 2015). Furthermore, the response of rose bitterling to snakehead increased with distances less than 20 cm but decreased profoundly beyond 20 cm (Hu, Nie & Fu, 2015). Thus, the effect of predator size on the distance maintained by potential prey requires further exploration.

### The effects of predators on the activities of prey

Interestingly, after introduction of the predator, the percent time moving of prey decreased approximately by 40% and 30%, whereas the total distance moved decreased by approximately 50% and 35%, in the size-matched predator and large predator treatments, respectively. The swimming speed while moving also showed approximately 12% and 10% decrease, although it was not statistically significant. However, black carp in the control treatment showed no change in any of measured activity variables before and after introduction of another black carp. This result suggests that the introduction of predators resulted in a reduction in the swimming activities of the prey fish. Similar phenomena have been frequently documented in previous studies. For example, predator attacks induced a 50% decrease in swimming activity in Atlantic salmon (*Salmo salar*) (Johnsson, Höjesjö & Fleming, 2001; Archard & Braithwaite, 2011), and the swimming activity decreased by 38% when three-spined stickleback were exposed to chemical and visual cues of their predators (Lehtiniemi, 2005). The underlying mechanism for decreased activities in black carp when exposed to snakehead might be the result of elevated anti-predation state, including increased vigilance and a decreased possibility of detection by predators. For example, redshank (*Tringa totanus*) individuals, which showed decreased time spent foraging in a high-risk environment, gained a survival advantage, and individuals with increased vigilance during foraging after seeing the predators (i.e., less feeding and slower to orientate or approach food items) had increased survival times (Metcalfe, Huntingford & Thorpe, 1987; Sansom, Lind & Cresswell, 2009). Moving coho salmon (*Oncorhynchus kisutch)* individuals were also easier to catch for their natural predators than individuals that stayed motionless (Martel & Dill, 1995). The A reduction in activity has been frequently demonstrated in aquatic animals, including fish species, as one of the main anti-predator strategies (Brown & Smith, 1998; Johnsson, Höjesjö & Fleming, 2001; Albecker & Vance-Chalcraft, 2015; Harding & Scheibling, 2015). Such adjustments in behavior in addition to maintaining a safe distance from predators might be crucial in the survival of prey in prey-predator interactions (Martel & Dill, 1995). It was interesting to find that there was no significant difference between two predator treatments in any of the variables involved in spontaneous activity either before or after the introduction of the predator. This suggests that predator size showed little effect on the behavior adjustments of black carp, which was similar to those of distance responses.

The percent time moving of fish in control group was significantly higher than those under size-matched predator treatment while there was no significant difference between control group and large predator treatment after introduction of a companion. This suggests that large size predator showed less impact on spontaneous behavior of black carp, which was unexpected because large predators theoretically have more profound psychological and behavioral effects on prey, and black carp should have decreased their activities more when near a large predator.

In conclusion, black carp showed longer distance from snakehead and less spontaneous activities after introduction of snakehead compared to those paired with their conspecifics. This suggests that black carp can recognize snakehead as a herterospecifics and probably recognize the snakehead as a potential predator, thus maintain a safe distance from the snakehead, increase its vigilance and decrease the exploration and foraging behaviors. However, no differences in safe distance and behavior adjustments were found between the size-matched and larger predator treatments, which suggested that the behaviors of the prey response to predators were not affected by relative size differences between them. It suggests that the main anti-predator strategy in black carp may involve maintaining a safe distance, decreased activity and possibly increased vigilance.

##  Supplemental Information

10.7717/peerj.3222/supp-1 Data S1 Raw data of the present study

## Additional Information and Declarations

### Data Availability

The following information was supplied regarding data availability: The raw data has been supplied as Data S1.
